# Comparing health care use and costs among new Medicaid enrollees before and during the COVID-19 pandemic

**DOI:** 10.1186/s12913-021-07027-6

**Published:** 2021-10-25

**Authors:** Brad Wright, David Anderson, Rebecca Whitaker, Peter Shrader, Janet Prvu Bettger, Charlene Wong, Paul Shafer

**Affiliations:** 1 Department of Family Medicine, University of North Carolina at Chapel Hill, 590 Manning Dr, NC CB 7595 Chapel Hill, United States; 2 Cecil G. Sheps Center for Health Services Research, University of North Carolina at Chapel Hill, Chapel Hill, USA; 3 Duke-Margolis Center for Health Policy, Washington, USA; 4 Duke Clinical Research Institute, Durham, USA; 5 Department of Orthopaedic Surgery, Duke University, Durham, USA; 6 Department of Pediatrics, Duke University, Durham, USA; 7 Department of Health Law, Policy, and Management, Boston University, Boston, USA

**Keywords:** Medicaid, COVID-19, North Carolina, Health Care Use, Costs

## Abstract

**Background and Objective:**

To characterize health care use and costs among new Medicaid enrollees before and during the COVID pandemic. Results can help Medicaid non-expansion states understand health care use and costs of new enrollees in a period of enrollment growth.

**Research Design:**

Retrospective cross-sectional analysis of North Carolina Medicaid claims data (January 1, 2018 - August 31, 2020). We used modified Poisson and ordinary least squares regression analysis to estimate health care use and costs as a function of personal characteristics and enrollment during COVID. Using data on existing enrollees before and during COVID, we projected the extent to which changes in outcomes among new enrollees during COVID were pandemic-related.

**Subjects:**

340,782 new enrollees pre-COVID (January 2018 – December 2019) and 56,428 new enrollees during COVID (March 2020 – June 2020).

**Measures:**

We observed new enrollees for 60-days after enrollment to identify emergency department (ED) visits, nonemergent ED visits, primary care visits, potentially-avoidable hospitalizations, dental visits, and health care costs.

**Results:**

New Medicaid enrollees during COVID were less likely to have an ED visit (-46 % [95 % CI: -48 %, -43 %]), nonemergent ED visit (-52 % [95 % CI: -56 %, -48 %]), potentially-avoidable hospitalization (-52 % [95 % CI: -60 %, -43 %]), primary care visit (-34 % [95 % CI: -36 %, -33 %]), or dental visit (-36 % [95 % CI: -41 %, -30 %]). They were also less likely to incur any health care costs (-29 % [95 % CI: -30 %, -28 %]), and their total costs were 8 % lower [95 % CI: -12 %, -4 %]. Depending on the outcome, COVID explained between 34 % and 100 % of these reductions.

**Conclusions:**

New Medicaid enrollees during COVID used significantly less care than new enrollees pre-COVID. Most of the reduction stems from pandemic-related changes in supply and demand, but the profile of new enrollees before versus during COVID also differed.

**Supplementary Information:**

The online version contains supplementary material available at 10.1186/s12913-021-07027-6.

## Introduction

The COVID-19 pandemic led to significant economic hardship and unemployment. Newly uninsured adults and their families were expected to enroll in Medicaid—the health insurance program for low-income Americans—and to enroll at higher rates in Medicaid expansion states than non-expansion states (i.e., states that have used the *Affordable Care Act*to expand Medicaid eligibility to all individuals with incomes up to 138 % of the federal poverty level versus states that have not) [1–3]. States braced for increased enrollment while responding to public health measures and limiting in-person health care services to prevent COVID-19 transmission. In North Carolina, a Medicaid non-expansion state, total Medicaid enrollment increased by 7.3 % (from 2,071,904 to 2,223,768 enrollees) between February 2020 and August 2020 [4]. However, the impact of this influx of new enrollees on health care use and costs is unclear. Understanding patterns of health care use and costs among new enrollees can provide states critical information for optimizing Medicaid programs.

When Medicaid expanded in Oregon in 2008 [5] and in other states via the Affordable Care Act (ACA) in 2014, questions arose about the health profile and use of new enrollees [6, 7], and the implications for state budgets. In Oregon, gaining Medicaid coverage was associated with increased use of primary and preventive care, emergency department care, and hospitalization during the first year of coverage [8]. By the second year, however, health care use had diminished to levels comparable to those continuously enrolled in Medicaid [9]. Moreover, approximately 40 % of new Medicaid enrollees in Oregon sought little to no care after gaining coverage either because they were relatively healthy or because they faced barriers ranging from confusion about their coverage to inadequate access to care [10].

Nationally, the Centers for Medicare and Medicaid Services (CMS) estimated that total health care costs—the costs borne by federal and state governments reimbursing providers for delivering health care services to Medicaid enrollees—would be 17 % higher among new Medicaid expansion-eligible enrollees compared to those who enrolled prior to Medicaid expansion [11]. However, health care costs among newly eligible and enrolled adults were actually 21 % lower than that of previously eligible enrollees ($228 vs. $180 per member-month), including significantly lower costs for both office-based and ED visits [12]. Other studies have found evidence of pent-up demand among new Medicaid enrollees in expansion states with use diminishing within a year or less of gaining coverage [6, 13].

These prior studies compared the health care use and cost patterns of new enrollees to those who were uninsured or continuously enrolled [7–9, 14, 15]. We add to the extant literature by comparing the health care use and cost patterns of individuals who were newly enrolled just prior to versus newly enrolled during the first wave of the COVID-19 pandemic. We use Medicaid data from North Carolina—the third most populous Medicaid non-expansion state—and focus on health care use and costs during the first 60 days of enrollment to capture the period when pent-up demand is likely to be at its highest [6, 13]. As the evolving impacts of the pandemic are being identified, our findings can help state Medicaid programs with resource allocation and budgetary considerations during a period of enrollment growth.

## Methods

We used North Carolina Medicaid claims data from January 1, 2018 through August 31, 2020 to examine new enrollees’ patterns of health care use and costs during their first 60 days of enrollment. We defined new Medicaid enrollees each month as individuals without Medicaid in the prior 6 months. We compared new enrollees who gained coverage in the pre-COVID period (January 2018 – December 2019) to new enrollees who gained coverage during COVID (March 2020 – June 2020). We excluded new enrollees in January and February 2020 to ensure that there was no overlap between the pre-COVID and COVID time periods. We used the most contemporaneous data available at the time (through August 2020) and considered new enrollees through the end of June 2020 so that we could observe the full 60-day follow up period. We received the data at the end of October 2020, ensuring a minimum of two months for claims runout.

We generated descriptive statistics on new Medicaid enrollees including age, sex, race, ethnicity, urbanicity, and Medicaid enrollment pathway. Then, we characterized a set of health care use and cost outcomes across our sample. These included five binary outcomes indicating whether the new enrollee had any emergency department (ED) visits, nonemergent ED visits (as defined by the NYU Algorithm) [16], primary care visits, potentially-avoidable hospitalizations (as defined by the Agency for Healthcare Research and Quality’s Prevention Quality Indicators) [17], or dental visits. We also calculated each of these outcomes as a rate, using the number of visits per 1,000 member-months. Additionally, we examined whether an enrollee had any health care costs, defined as all costs reimbursed to providers by the Medicaid program, as well as a continuous measure of their total costs per member-month, conditional on them having any health care costs. Together, these outcomes allow us to understand how new enrollees used the health care system immediately after gaining Medicaid coverage. We stratified all bivariate analyses by the pre-COVID and COVID periods. Additionally, we stratified the COVID period by month to examine trends over time during the pandemic (see Appendix Table 1 in Additional File 1). We also examined trends in our outcomes for all new enrollees by month (see Appendix Figs. 1, 2, 3, 4, 5, 6, 7, 8, 9, 10, 11 and 12 in Additional File 1).

**Table 1:** Characteristics of New Medicaid Enrollees Pre-COVID and New Enrollees During COVID, North Carolina

	1/2018–12/2019	3/2020–6/2020	P
Number of new enrollees	340,782	56,428	
Age at enrollment, years			< 0.0001
Median (Q1, Q3)	38.6 (28.4, 55.6)	36.8 (28.1, 51.6)	
Mean (SD)	42.9 (17.6)	41.1 (16.6)	
Range	18.0, 115.3	18.0, 106.3	
Sex			0.0009
Female	227,060 (66.6 %)	37,197 (65.9 %)	
Male	113,722 (33.4 %)	19,231 (34.1 %)	
Race			< 0.0001
White	213,335 (62.6 %)	34,654 (61.4 %)	
Black	104,827 (30.8 %)	17,806 (31.6 %)	
Multiple/Other	19,853 (5.8 %)	3,209 (5.7 %)	
Unknown	2,767 (0.8 %)	759 (1.3 %)	
Ethnicity			< 0.0001
Hispanic/Latinx	44,545 (13.1 %)	6,974 (12.4 %)	
Non-Hispanic/Latinx	285,727 (83.8 %)	47,707 (84.5 %)	
Unknown	10,510 (3.1 %)	1,747 (3.1 %)	
Location			
Urban	229,226 (67.3 %)	42,540 (75.4 %)	< 0.0001
Rural	111,556 (32.7 %)	13, 888 (24.6 %)	
Enrollment pathway			< 0.0001
Dual-Eligibles	88,170 (25.9 %)	8,528 (15.1 %)	
Pregnant Women & Breast / Cervical Cancer Program	38,620 (11.3 %)	5,958 (10.6 %)	
Low-Income Adult	181,640 (53.3 %)	37,001 (65.6 %)	
General Pediatrics	12,507 (3.7 %)	2,073 (3.7 %)	
Other	19,845 (5.8 %)	2,815 (5.0 %)	

Then, we modeled our binary outcomes using modified Poisson regression analyses to directly estimate relative risks[18], as shown in Eq. 1:

1 $${\text{l}\text{o}\text{g}(Y}_{i})=\alpha +({\beta }_{1a}{T}_{2i}{+\dots +\beta }_{1k}{T}_{12i})+{\beta }_{2}{Post}_{i}+ {\beta }_{3}{\boldsymbol{X}}_{i}+ {\epsilon }_{i}$$

where Y represents our outcomes of interest for individual *i*, *T*_*1,…,12*_ represents a set of calendar month fixed effect indicators for the month of Medicaid enrollment to account for seasonality (omitting January as the reference month), *Post* is an indicator of the COVID period (beginning March 1, 2020), and ***X*** is a vector of enrollee characteristics including age, sex, race, ethnicity, and rurality that are likely to be associated with our outcomes because of predisposing, enabling, and need factors [19, 20] as well as systemic factors like institutional and interpersonal racism [21], all of which influence access to and use of health care. The coefficient ${\beta }_{2}$ captures the difference in outcomes between new enrollees in the pre-COVID and COVID periods. To assess the extent to which this coefficient is dependent on demographic differences between enrollees in the two periods, we also estimated unadjusted models (see Appendix Tables 2 and 3 in Additional File 1).

**Table 2:** Modified Poisson Regression Results of Health Care Use among New Medicaid Enrollees Pre-COVID and New Enrollees During COVID, North Carolina

	*Any ED*		*Any* *Nonemergent ED*		*Any Potentially-* *avoidable Hospitalization*		*Any Primary care*		*Any Dental*	
*Covariate*	*RR*	*(95 % CI)*	*RR*	*(95 % CI)*	*RR*	*(95 % CI)*	*RR*	*(95 % CI)*	*RR*	*(95 % CI)*
Enrollment Month										
January	Reference		Reference		Reference		Reference		Reference	
February	1.01	(0.96–1.05)	1.01	(0.92–1.12)	0.87	(0.71–1.07)	1.04*	(1.01–1.07)	1.06	(0.95–1.18)
March	0.78***	(0.74–0.81)	0.77***	(0.70–0.84)	0.73**	(0.61–0.88)	0.82***	(0.80–0.85)	0.73***	(0.66–0.81)
April	0.90***	(0.86–0.94)	0.92	(0.84–1.01)	0.76**	(0.62–0.93)	0.94***	(0.92–0.97)	0.91	(0.82–1.02)
May	0.95*	(0.91–0.99)	0.92	(0.84–1.02)	0.91	(0.75–1.10)	0.95**	(0.93–0.98)	0.88*	(0.79–0.98)
June	0.93**	(0.89–0.97)	0.98	(0.89–1.07)	0.79*	(0.65–0.97)	0.94***	(0.91–0.97)	1.05	(0.95–1.16)
July	0.89***	(0.85–0.94)	0.90*	(0.82–0.99)	0.82	(0.66-1.00)	0.91***	(0.88–0.94)	0.96	(0.86–1.07)
August	0.85***	(0.81–0.89)	0.88**	(0.80–0.97)	0.86	(0.70–1.05)	0.88***	(0.86–0.91)	0.90	(0.81–1.01)
September	0.93**	(0.89–0.98)	0.93	(0.84–1.03)	0.77*	(0.61–0.95)	1.01	(0.98–1.04)	1.13*	(1.02–1.27)
October	0.89***	(0.85–0.93)	0.90*	(0.81–0.99)	0.70	(0.56–0.88)	0.98	(0.95–1.01)	0.99	(0.88–1.11)
November	0.79***	(0.75–0.84)	0.79***	(0.71–0.88)	0.87	(0.70–1.08)	0.84***	(0.81–0.87)	0.76***	(0.67–0.86)
December	0.95	(0.90-1.00)	0.87*	(0.77–0.97)	0.90	(0.71–1.14)	0.92***	(0.89–0.96)	0.98	(0.86–1.12)
Enrolled During COVID	0.54***	(0.52–0.57)	0.48***	(0.44–0.52)	0.48***	(0.40–0.57)	0.66***	(0.64–0.67)	0.64***	(0.59–0.70)
Age at enrollment										
18–25	Reference		Reference		Reference		Reference		Reference	
26–35	0.84***	(0.82–0.86)	0.91***	(0.86–0.96)	1.18*	(1.00-1.38)	0.92***	(0.90–0.93)	1.01	(0.95–1.08)
36–45	0.76***	(0.73–0.78)	0.86***	(0.81–0.92)	1.75***	(1.49–2.05)	0.71***	(0.69–0.72)	0.98	(0.91–1.05)
46–55	0.66***	(0.64–0.69)	0.80***	(0.74–0.85)	2.01***	(1.71–2.37)	0.58***	(0.56–0.60)	0.64***	(0.58–0.70)
56–65	0.59***	(0.57–0.61)	0.71***	(0.65–0.76)	1.79***	(1.51–2.12)	0.62***	(0.61–0.64)	0.50***	(0.45–0.55)
65+	0.29***	(0.28–0.31)	0.36***	(0.32–0.40)	1.09	(0.89–1.33)	0.42***	(0.41–0.44)	0.54***	(0.49–0.59)
Race										
White	Reference		Reference		Reference		Reference		Reference	
Black	1.21***	(1.18–1.23)	1.37***	(1.31–1.43)	1.31***	(1.19–1.44)	0.89***	(0.88–0.91)	1.14***	(1.08–1.20)
Multiple/Other	0.67***	(0.64–0.71)	0.65***	(0.58–0.73)	0.58***	(0.45–0.75)	0.74***	(0.71–0.76)	0.72***	(0.64–0.80)
Unknown	0.52***	(0.44–0.61)	0.77*	(0.59-1.00)	0.69	(0.41–1.17)	0.55***	(0.50–0.61)	0.83	(0.60–1.14)
Ethnicity										
Non-Hispanic	Reference		Reference		Reference		Reference		Reference	
Hispanic	0.97	(0.94–1.01)	0.75***	(0.70–0.81)	0.98	(0.84–1.15)	1.81***	(1.78–1.84)	0.76***	(0.71–0.83)
Unknown	1.47***	(1.39–1.55)	1.82***	(1.65–2.01)	2.20***	(1.83–2.64)	1.49***	(1.43–1.55)	1.05	(0.90–1.22)
Live in a rural county	1.22***	(1.19–1.24)	1.19***	(1.14–1.24)	1.23***	(1.11–1.35)	1.11***	(1.09–1.12)	1.13***	(1.07–1.19)
Female	1.16***	(1.13–1.19)	1.13***	(1.08–1.18)	0.69***	(0.63–0.75)	1.81***	(1.78–1.85)	1.07**	(1.02–1.13)
	N = 360,508		N = 360,508		N = 360,508		N = 360,508		N = 360.508	

*p < 0.05 **p < 0.01 ***p < 0.001

**Table 3:** Two-Part Model Results of Having Any Health Care Costs and Per Member Per Month Cost Models among New Medicaid Enrollees Pre-COVID and New Enrollees During COVID, North Carolina

*Covariate*	*Any Cost Accrued*		*Per Member Per Month Cost, Exponentiated Beta (SE)*
Enrollment Month	*RR*	*(95 % CI)*	
January	Reference		Reference
February	1.02	(1.00-1.04)	1.02 (1.03)
March	0.83***	(0.81–0.84)	1.07** (1.02)
April	0.93***	(0.92–0.95)	1.02 (1.02)
May	0.96***	(0.95–0.98)	1.03 (1.02)
June	0.95***	(0.93–0.97)	1.01 (1.03)
July	0.93***	(0.91–0.95)	1.02 (1.03)
August	0.89***	(0.87–0.91)	0.96 (1.03)
September	0.97**	(0.95–0.99)	1.04 (1.03)
October	0.93***	(0.92–0.95)	0.98 (1.03)
November	0.85***	(0.83–0.87)	0.99 (1.03)
December	0.98*	(0.96-1.00)	1.12*** (1.03)
Enrolled During COVID	0.71***	(0.70–0.72)	0.92*** (1.02)
Age at enrollment			
18–25	Reference		Reference
26–35	0.91***	(0.90–0.92)	1.28*** (1.01)
36–45	0.72***	(0.71–0.73)	1.67*** (1.02)
46–55	0.56***	(0.55–0.57)	1.95*** (1.02)
56–64	0.59***	(0.58–0.61)	1.63*** (1.02)
65+	0.84***	(0.82–0.85)	2.24*** (1.02)
Race			
White	Reference		Reference
Black	0.92***	(0.91–0.93)	0.90*** (1.01)
Other	0.70***	(0.69–0.72)	0.86*** (1.03)
Unknown	0.55***	(0.51–0.59)	0.50*** (1.08)
Ethnicity			
Non-Hispanic	Reference		Reference
Hispanic	1.53***	(1.51–1.54)	2.60*** (1.01)
Unknown	1.18***	(1.14–1.21)	1.04 (1.03)
Live in a rural county	1.12***	(1.11–1.13)	0.94*** (1.01)
Female	1.61***	(1.59–1.63)	0.71*** (1.01)
	N = 360,508		N = 126,429

*p < 0.05 **p < 0.01 ***p < 0.001

Note: Any cost accrued is modeled using modified Poisson regression, while the per member per month cost (conditional on having non-zero costs) is modeled using ordinary least squares regression.

To examine health care costs, we used a two-part model, because a significant proportion of new enrollees had no health care costs. The first stage examined the binary outcome of having any health care costs using modified Poisson regression as shown in Eq. 1. The second stage used an ordinary least squares model with the same covariates to estimate the amount of health care costs an individual had, conditional on having non-zero health care costs. We logged our outcome because health care cost data are heavily skewed. We did not adjust costs for inflation as the relevant period was too short for inflation to be meaningful. We could not include enrollment pathway as a covariate in our main models because of its highly correlated, and in some cases deterministic, relationship with other characteristics (e.g., pregnancy and sex). Thus, we also ran stratified versions of all models outlined above by enrollment pathway, including both unadjusted and adjusted results (see Appendix Tables 4, 5 and 6 in Additional File 1).

**Table 4:** Bivariate Analysis of Actual Outcomes (Health Care Use and Costs) among New Medicaid Enrollees Pre-COVID and Actual and Projected Outcomes for New Enrollees During COVID, North Carolina

Outcome	Pre-COVID		During COVID		
	*Existing* *Enrollees*	*New Enrollees*	*Existing* *Enrollees*	*New Enrollees (projected)*^*a*^	*New Enrollees (actual)*
At least one ED visit	393,324 (19.5 %)	36,794 (10.8 %)	120,824 (12.9 %)	4,006 (7.1 %)	3,315 (5.9 %)
ED visits per 1,000 member-months (95 % CI)	113.38 (113.19-113.58)	119.59 (118.75-120.43)	67.65 (67.43–67.87)	71.36 (69.89–72.83)	61.55 (60.08–63.05)
At least one nonemergent ED visit	167,672 (8.3 %)	9,361 (2.7 %)	47,329 (5.1 %)	959 (1.7 %)	756 (1.3 %)
Nonemergent ED visits per 1,000 member-months (95 % CI)	24.01 (23.93–24.10)	18.89 (18.55–19.22)	13.53 (13.44–13.63)	10.64 (10.01–11.27)	9.40 (8.84-10.00)
At least one potentially-avoidable hospitalization	36,961 (1.8 %)	2,143 (0.6 %)	10,873 (1.2 %)	226 (0.4 %)	161 (0.3 %)
Potentially-avoidable hospitalizations per 1,000 member-months (95 % CI)	4.53 (4.49–4.57)	4.08 (3.93–4.24)	2.76 (2.72–2.81)	2.49 (2.22–2.76)	1.90 (1.66–2.18)
At least one primary care visit	890,962 (44.1 %)	60,954 (17.9 %)	333,940 (35.6 %)	8,126 (14.4 %)	6,508 (11.5 %)
Primary care visits per 1,000 member-months (95 % CI)	322.90 (322.57-323.23)	188.23 (187.18–189.30)	196.39 (196.03-196.76)	114.48 (112.50-116.46)	110.81 (108.84-112.81)
At least one dental visit	296,051 (14.7 %)	6,720 (2.0 %)	86,951 (9.3 %)	734 (1.3 %)	741 (1.3 %)
Dental visits per 1,000 member-months (95 % CI)	50.42 (50.29–50.55)	17.90 (17.58–18.23)	26.61 (26.47–26.75)	9.45 (8.81–10.09)	10.95 (10.34–11.59)
Subjects with some costs	1,159,347 (57.4 %)	125,061 (36.7 %)	480,835 (51.3 %)	18,508 (32.8 %)	14,239 (25.2 %)
Total cost in dollars per member-months (95 % CI)	380.40 (380.04-380.75)	500.98 (499.26-502.71)	336.64 (336.16-337.12)	443.35 (440.05-446.65)	302.62 (299.36-305.93)
N	2,020,253	340,782	936,870	56,428	56,428

Note: All differences between time periods within enrollee groups are significant at *P* < 0.0001

^a^ These projections were calculated by taking the ratio of during COVID and pre-COVID existing enrollee outcomes and multiplying it by the pre-COVID new enrollee outcomes

To account for large and widespread reductions in health care use and costs in the early months of the pandemic resulting from both health care providers limiting non-essential care and patients voluntarily foregoing care to avoid contracting COVID, we generated back-of-the-envelope projections for each outcome among new enrollees in the COVID period as shown in Eq. 2:

2 $$Projected New {Enrollees}_{COVID}=\left(Observed New {Enrollees}_{Pre-COVID}\right)\left(\frac{Observed Existing {Enrollees}_{COVID}}{Observed Existing {Enrollees}_{Pre-COVID}}\right)$$

In effect, this creates a COVID adjustment factor to estimate declines in service use and costs during the pandemic. Comparing the observed outcomes among new enrollees in the COVID period to these projections allows us to decompose the observed differences among new enrollees pre-COVID and new enrollees during COVID into two components: (1) COVID-related reductions in the ability and/or willingness to access care, and (2) residual differences in composition (i.e., systematic differences between new enrollees in the pre-COVID and COVID periods).

## Results

Characteristics of the new Medicaid enrollee population are shown in Table 1, stratified by pre-COVID and COVID time periods. New enrollees during COVID are younger and more likely to be male, Black, non-Hispanic, and live in urban areas. New enrollees during COVID are also considerably more likely to qualify for Medicaid through the low-income adult pathway (65.6 % vs. 53.3 %).

Table 2 presents the results of our multivariable regression models. Among new Medicaid enrollees during COVID, we found reductions in the probability of having an ED visit (-46 % [95 % CI: -48 %, -43 %]), a nonemergent ED visit (-52 % [95 % CI: -56 %, -48 %]), a potentially-avoidable hospitalization (-52 % [95 % CI: -60 %, -43 %]), a primary care visit (-34 % [95 % CI: -36 %, -33 %]), a dental visit (-36 % [95 % CI: -41 %, -30 %]), and incurring any health care costs within the first 60 days of coverage (-29 % [95 % CI: -30 %, -28 %], Table 3) compared to new enrollees pre-COVID. These results did not vary significantly in our unadjusted models, suggesting that differences in observable characteristics of new enrollees between the two time periods did not drive our findings (see Appendix Tables 2 and 3, in Additional File 1).

Across the entire study period, we also found significant differences by age, sex, race, ethnicity, and rurality. These are overall differences, not a comparison before and during COVID. Compared to enrollees ages 18–25, we found that the probability of having an ED visit, nonemergent ED visit, primary care visit, or dental visit generally decreased with increasing age, while the probability of experiencing a potentially-avoidable hospitalization increased. Women were nearly twice as likely as men to use primary care and were 61 % more likely to incur any health care costs ([95 % CI: 59 %, 63 %], Table 3). As shown in Table 2, they also had a higher probability of visiting the ED for emergent and nonemergent care and receiving dental care but were 31 % less likely to have a potentially-avoidable hospitalization [95 % CI: -37 %, -25 %]. Compared to White enrollees, Black enrollees were more likely to have an ED visit (21 % [95 % CI: 18 %, 23 %]), nonemergent ED visit (37 % [95 % CI: 31 %, 43 %]), potentially-avoidable hospitalization (31 % [95 % CI: 19 %, 44 %]), or dental visit (14 % [95 % CI: 8 %, 20 %]), but less likely to have a primary care visit (-11 % [95 % CI: -12 %, -9 %]) or incur any health care costs (-8 % [95 % CI: -9 %, -7 %], Table 3). Hispanic enrollees had a lower probability of nonemergent ED (-25 % [95 % CI: -30 %, -19 %]) and dental visits (-24 % [95 % CI: -29 %, -17 %]), but a much higher probability of having a primary care visit (81 % [95 % CI: 78 %, 84 %]) or incurring any health care costs (53 % [95 % CI: 51 %, 54 %], Table 3) compared to non-Hispanic enrollees. Finally, across all our outcomes, we found a higher probability of health care use and costs among rural versus urban enrollees.

Table 3 presents the results of our two-part model of health care costs per member-month during the first 60 days of enrollment. On average, we did not observe a strong temporal trend, although costs were 7 % higher for March enrollees [95 % CI: 3.1 %, 10.9 %] and 12 % higher for December enrollees [95 % CI: 6.1 %, 17.9 %] compared to January enrollees. We found that costs were 8 % lower on average during COVID [95 % CI: -12 %, -4 %]. This result did not change in our unadjusted model (see Appendix Table 3, in Additional File 1^1^). We also found that costs are 29 % lower for women versus men during their first 60 days enrolled [95 % CI: -31 %, -27 %], 6 % lower for rural residents versus urban residents [95 % CI: -8 %, -4 %], 10–14 % lower for non-White versus White enrollees, and 160 % higher for Hispanic versus non-Hispanic enrollees [95 % CI: 158 %, 162 %]. Despite finding that the probability of health care use tended to decline with age among new enrollees, we also found that among those who do use care, costs increase steadily with age, more than doubling among the 65 and older population compared to those ages 18–25.

The results of our back-of-the-envelope effort to quantify the extent to which the reduction in health care use and costs we identify is a result of a sizable pandemic-related shock to health care use and costs are shown in Table 4. These projections represent the amount of health care use and costs we would anticipate among new enrollees during COVID based on the levels of health care use and costs among new enrollees in the pre-COVID period, after applying a pandemic-related adjustment derived from changes in health care use and costs we observe among existing enrollees. Comparing the observed and projected values for new enrollees during COVID, we found that these individuals still had lower than projected levels of health care use and costs even after adjusting for widespread access reductions due to the pandemic, except for dental care. For example, new enrollees during COVID averaged 61.6 ED visits and 110.8 primary care visits per 1,000 member-months. Pre-COVID, new enrollees averaged 119.6 ED visits and 188.2 primary care visits per 1,000 member-months. By our calculations, the “COVID effect” would lead to a projected 71.4 ED visits [95 % CI: 69.9, 72.8] and 114.5 primary care visits [95 % CI: 112.5, 116.5] per 1,000 member-months among new enrollees during COVID. Thus, we attribute the remaining 9.8 fewer ED visits and 3.7 fewer primary care visits per 1,000 member-months to differences in the new enrollee populations between the pre-COVID and COVID periods. In examining the new enrollee data during COVID by month, we noted a steady decrease in nonemergent ED visits and a steady increase in the use of dental care from March to June of 2020 (see Appendix Table 1, in Additional File 1).

## Discussion

In this analysis of health care use and costs among new Medicaid enrollees before and during the COVID-19 pandemic, we found that new enrollees during COVID were significantly less likely to use primary care, visit the ED or dentist, experience potentially-avoidable hospitalization, or incur any health care costs compared to new enrollees before the pandemic even after accounting for COVID-era reductions in health care use. We also found that among new enrollees who had health care costs within their first 60 days of coverage, costs per member-month were lower for new enrollees during COVID than for new enrollees pre-COVID. Our findings have important implications for North Carolina and the 11 other states that have yet to expand Medicaid by helping them to anticipate estimated costs and health care use for individuals who newly enrolled during the COVID pandemic.

The lower rates of health care use among new Medicaid enrollees during COVID suggest two possibilities. First, the pandemic led to a rapid decrease in all health care use as people elected to forego care during the first several months of the pandemic to avoid possible exposure to COVID, and some health care facilities closed temporarily or pivoted to telehealth. Second, new Medicaid enrollees during COVID may differ in their health care needs and demand compared to new enrollees pre-COVID. For example, if new enrollees during COVID had employer-sponsored insurance prior to losing their jobs due to the economic impacts of COVID and became eligible for Medicaid, they may have had less pent-up demand for care. Our bivariate analyses (Table 4) suggest the first reason had the stronger effect since we used changes in health care use and costs among existing enrollees to anticipate what health care use and costs would have been among new enrollees during COVID. Here we found that COVID accounted for 100 % of the reduction in the probability of having a dental visit, 76 % of the reduction in the probability of having an ED visit, 71 % of the reduction in the probability of having a nonemergent ED visit, 67 % of the reduction in the probability of having a potentially-avoidable hospitalization, 55 % of the reduction in the probability of having a primary care visit, and 34 % of the reduction in the probability of having any health care costs. The remaining proportion of the reduction in these outcomes can be attributed to compositional differences between the new enrollee groups before and during COVID.

Lower utilization rates were accompanied by findings of lower cost during COVID. States are rightfully concerned about the impact of Medicaid on their budgets, which they must balance each year [22]. Concerns are compounded because of increased enrollment that typically occurs during economic downturns when revenues to fund the program decline [23]. One solution proposed by the Medicaid and CHIP Payment and Access Commission is to amend the *Social Security Act*to create an automatic countercyclical financing adjustment that would temporarily increase the federal government’s share of Medicaid costs during economic downturns [24]. This would lessen the budgetary pressure on states that can result in cuts to optional Medicaid benefits and enrollment, helping to ensure that people do not lose their health insurance coverage during an economic crisis. Our results suggest that the costs of such a program might be less than anticipated.

Across both time periods, we found notable associations among new Medicaid enrollees generally (i.e., not a before and during COVID comparison). While only 24.7 % of new enrollees in the pre-COVID period and 15.8 % of new enrollees during COVID had at least one claim for our outcomes of interest within their first 60 days of coverage, younger enrollees, women, and enrollees living in rural areas are more likely to rely on their Medicaid coverage soon after enrollment. Moreover, our finding that new enrollees who are Black are less likely to use primary care and more likely to use the ED and experience potentially-avoidable hospitalizations reflects previous studies [25–33] and underscores the structural inequities in access to care and poorer health outcomes resulting from systemic racism [21, 34–42]. The COVID-19 pandemic is an extreme example of a time when Medicaid is even more vital than usual for marginalized populations. Recognizing this, the *Families First Coronavirus Response Act*instituted maintenance of effort requirements that prevent states from terminating eligibility or benefits during the national public health emergency, while also temporarily increasing the federal government’s share of Medicaid expenses to alleviate the financial burden on states [43].

Overall, we found that new Medicaid enrollees during the COVID-19 pandemic used significantly less care than new enrollees in the two preceding calendar years. With the exception of having any health care costs, most of this reduction can be attributed to the pandemic itself as a combination of the reduced demand for health care services from individuals concerned about contracting COVID and the reduced supply of health care services as hospitals, clinics and other medical providers restructured care to prioritize COVID-19 and enact public health safety measures. Differences in the profile of new enrollees during COVID versus pre-pandemic, likely also played a role in the reductions in health care use and costs we observed.

Our study is subject to some limitations. First, the cross-sectional design limits our ability to draw causal inferences from the differences we observe between new enrollees in the pre-COVID and COVID periods. Second, because of the timeliness of our study, we are limited to only 6 months of claims data during COVID. To maximize both the number of new enrollees in our study and the length of time we observe them after they enroll, we only followed new enrollees for 60 days after enrollment. While this is likely to capture pent-up demand, we recognize—and our data indicate—that only a minority of new enrollees use health care soon after enrollment. With more data, we would gain a more complete picture of the health care use and costs of new enrollees—particularly in the COVID period. Relatedly, we are still amid the pandemic, so health care use and costs continue to exhibit atypical fluctuations, and without observing a full 12 months during COVID, there may be additional COVID-specific seasonality for which we are unable to fully adjust. In using the most contemporaneous data available, it is also possible that we are missing some claims during COVID that had yet to be fully adjudicated. However, with at least two months of claims runout for even the newest enrollees represented in our data, we expect this concern to be minimal. Finally, North Carolina data may not generalize to other (particularly Medicaid expansion) states. Had North Carolina expanded Medicaid, we expect that we would have observed many more new enrollees during the COVID period, but it is unclear how the different demographic and health profile of the expansion population might have changed our findings.

Understandably, some may question what will happen to health care use and costs once pandemic-related restrictions are relaxed and the supply and demand for care return to near pre-pandemic levels. While we can only speculate, we would anticipate that both health care use and costs would increase on a per member-month basis, but that this would be offset in the aggregate by declines in Medicaid enrollment as the economy and employment recover. That said, given the enhanced federal matching rate and our finding that newly enrolled individuals during COVID are exhibiting less pent-up demand than new enrollees pre-COVID, Medicaid expansion would clearly benefit the state and its residents, particularly during a pandemic-induced recession or future public health and economic crises.

## Supplementary Information

**Additional file 1:** Appendix **Table S1**. Bivariate Analysis of Health Care Use and Costs among New Medicaid Enrollees During COVID, by Month, North Carolina Appendix **Table S2**. Unadjusted Modified Poisson Regression Results of Health Care Use among New Medicaid Enrollees Pre-COVID and New Medicaid Enrollees During COVID, North Carolina Appendix **Table S3**. Unadjusted Two-Part Model Results of Having Any Health Care Costs and Per Member Per Month Cost Models among New Medicaid Enrollees Pre-COVID and New Enrollees During COVID, North Carolina Appendix **Table S4**. Unadjusted Models of Health Care Use and Costs of New North Carolina Medicaid Enrollees During COVID Compared to New Enrollees Pre-COVID, Stratified by Eligibility Pathway Appendix **Table S5**. Adjusted Models of Health Care Use and Costs of New North Carolina Medicaid Enrollees During COVID Compared to New Enrollees Pre-COVID, Stratified by Eligibility Pathway Appendix **Table S6**. Unadjusted and Adjusted Two-Part Model Results of Per Member Per Month Cost Models of New North Carolina Medicaid Enrollees During COVID Compared to New Enrollees Pre-COVID, Stratified by Eligibility Pathway Figures S1 – S12. Monthly Trends in Health Care Use and Costs among New North Carolina Medicaid Enrollees Before and During COVID

## Data Availability

The data for this study were made available to us under an agreement with the North Carolina Department of Health and Human Services and cannot be shared. If someone wants to request the data from this study, they should contact the North Carolina Department of Health and Human Services, North Carolina Medicaid Division of Health Benefits (Mailing address: 2501 Mail Service Center, Raleigh, NC 27,699 − 2501; Phone: 1-888-245-0179).
