# Supplementary material for: Comparing health care use and costs among new Medicaid enrollees before and during the COVID-19 pandemic
Source: BMC Health Serv Res. 2021 Oct 25;21:1152. doi: 10.1186/s12913-021-07027-6 (PMC8544632; doi:10.1186/s12913-021-07027-6)
Supplement: Supplementary file 1 — Additional file 1: Appendix Table S1. Bivariate Analysis of Health Care Use and Costs among New Medicaid Enrollees During COVID, by Month, North Carolina Appendix Table S2. Unadjusted Modified Poisson Regression Results of Health Care Use among New Medicaid Enrollees Pre-COVID and New Medicaid Enrollees During COVID, North Carolina Appendix Table S3. Unadjusted Two-Part Model Results of Having Any Health Care Costs and Per Member Per Month Cost Models among New Medicaid Enrollees Pre-COVID and New Enrollees During COVID, North Carolina Appendix Table S4. Unadjusted Models of Health Care Use and Costs of New North Carolina Medicaid Enrollees During COVID Compared to New Enrollees Pre-COVID, Stratified by Eligibility Pathway Appendix Table S5. Adjusted Models of Health Care Use and Costs of New North Carolina Medicaid Enrollees During COVID Compared to New Enrollees Pre-COVID, Stratified by Eligibility Pathway Appendix Table S6. Unadjusted and Adjusted Two-Part Model Results of Per Member Per Month Cost Models of New North Carolina Medicaid Enrollees During COVID Compared to New Enrollees Pre-COVID, Stratified by Eligibility Pathway Figures S1 – S12. Monthly Trends in Health Care Use and Costs among New North Carolina Medicaid Enrollees Before and During COVID [file 12913_2021_7027_MOESM1_ESM.docx]

**Appendix Table 1. Bivariate Analysis of Health Care Use and Costs among New Medicaid Enrollees During COVID, by Month, North Carolina**

| **Outcome** | **Overall** | **March 2020** | **April 2020** | **May 2020** | **June 2020** |
| --- | --- | --- | --- | --- | --- |
| Number of enrollees | 56,428 | 14,824 | 16,592 | 12,264 | 12,748 |
|  |  |  |  |  |  |
| At least one ED visit | 3,315 (5.9%) | 1,094 (7.4%) | 824 (5.0%) | 740 (6.0%) | 657 (5.2%) |
| ED visits per 1,000 member-months | 61.55 (60.08-63.05) | 78.96 (75.75-82.31) | 51.18 (48.76-53.72) | 62.47 (59.34-65.77) | 54.03 (51.19-57.03) |
|  |  |  |  |  |  |
| At least one non-emergent ED visit | 756 (1.3%) | 261 (1.8%) | 199 (1.2%) | 143 (1.2%) | 153 (1.2%) |
| Non-emergent ED visits per 1,000 member-months | 9.40 (8.84-10.00) | 12.53 (11.29-13.90) | 8.53 (7.57-9.61) | 8.38 (7.28-9.64) | 7.88 (6.84-9.08) |
|  |  |  |  |  |  |
| At least one potentially-avoidable hospitalization | 161 (0.3%) | 60 (0.4%) | 36 (0.2%) | 40 (0.3%) | 25 (0.2%) |
| Potentially-avoidable hospitalizations per 1,000 member-months | 1.90 (1.66-2.18) | 2.80 (2.24-3.49) | 1.35 (1.00-1.82) | 2.11 (1.59-2.79) | 1.40 (1.00-1.95) |
|  |  |  |  |  |  |
| At least one primary care visit | 6,508 (11.5%) | 1,860 (12.5%) | 1,741 (10.5%) | 1,476 (12.0%) | 1,431 (11.2%) |
| Primary care visits per 1,000 member-months | 110.81 (108.84-112.81) | 124.05 (120.01-128.23) | 101.83 (98.38-105.39) | 116.01 (111.72-120.47) | 102.23 (98.29-106.32) |
|  |  |  |  |  |  |
| At least one dental visit | 741 (1.3%) | 102 (0.7%) | 206 (1.2%) | 190 (1.5%) | 243 (1.9%) |
| Dental visits per 1,000 member-months | 10.95 (10.34-11.59) | 5.45 (4.65-6.38) | 10.29 (9.23-11.46) | 13.49 (12.08-15.07) | 15.77 (14.26-17.42) |
|  |  |  |  |  |  |
| Subjects with some costs | 14,239 (25.2%) | 4,219 (28.5%) | 3,729 (22.5%) | 3,158 (25.8%) | 3,133 (24.6%) |
| Total cost in dollars per 1 member-month | 302.62 (299.36-305.93) | 379.37 (372.26-386.62) | 268.09 (262.46-273.83) | 304.19 (297.18-311.35) | 257.31 (251.02-263.76) |

**Appendix Table 2. Unadjusted Modified Poisson Regression Results of Health Care Use among New Medicaid Enrollees Pre-COVID and During COVID, North Carolina**

|  | *Any ED* | | *Any*  *Nonemergent ED* | | *Any Potentially-*  *avoidable Hospitalization* | | *Any Primary care* | | *Any Dental* | |
| --- | --- | --- | --- | --- | --- | --- | --- | --- | --- | --- |
| *Covariate* | *RR* | *(95% CI)* | *RR* | *(95% CI)* | *RR* | *(95% CI)* | *RR* | *(95% CI)* | *RR* | *(95% CI)* |
| Enrollment Month |  |  |  |  |  |  |  |  |  |  |
| January | Reference | | Reference | | Reference | | Reference | | Reference | |
| February | 1.01 | (0.96-1.06) | 1.02 | (0.93-1.12) | 0.86 | (0.71-1.05) | 1.04* | (1.00-1.07) | 1.06 | (0.95-1.18) |
| March | 0.79*** | (0.76-0.82) | 0.79*** | (0.73-0.87) | 0.72*** | (0.60-0.87) | 0.80*** | (0.78-0.83) | 0.74*** | (0.66-0.82) |
| April | 0.90*** | (0.86-0.94) | 0.93 | (0.85-1.02) | 0.77** | (0.63-0.93) | 0.92*** | (0.89-0.96) | 0.92 | (0.82-1.02) |
| May | 0.95* | (0.91-0.99) | 0.92 | (0.84-1.01) | 0.90 | (0.75-1.08) | 0.95** | (0.92-0.99) | 0.88* | (0.79-0.98) |
| June | 0.94** | (0.90-0.98) | 0.97 | (0.88-1.06) | 0.77** | (0.63-0.94) | 0.94*** | (0.91-0.97) | 1.05 | (0.95-1.17) |
| July | 0.92*** | (0.88-0.96) | 0.90* | (0.82-0.99) | 0.83 | (0.69-1.01) | 0.93*** | (0.90-0.96) | 0.96 | (0.86-1.07) |
| August | 0.88*** | (0.84-0.92) | 0.90* | (0.82-0.99) | 0.83 | (0.69-1.01) | 0.90*** | (0.87-0.93) | 0.89* | (0.80-1.00) |
| September | 0.94** | (0.90-0.98) | 0.92 | (0.83-1.01) | 0.74** | (0.60-0.92) | 1.00 | (0.97-1.04) | 1.08 | (0.97-1.20) |
| October | 0.92*** | (0.88-0.96) | 0.88* | (0.80-0.97) | 0.72** | (0.59-0.88) | 0.96* | (0.93-0.99) | 0.90 | (0.81-1.01) |
| November | 0.82*** | (0.79-0.86) | 0.80*** | (0.72-0.88) | 0.90 | (0.74-1.09) | 0.78*** | (0.75-0.81) | 0.63*** | (0.56-0.72) |
| December | 0.89 | (0.85-0.93) | 0.82*** | (0.74-0.90) | 0.86 | (0.70-1.04) | 0.83*** | (0.80-0.86) | 0.71*** | (0.62-0.80) |
|  |  |  |  |  |  |  |  |  |  |  |
| Enrolled During COVID | 0.55*** | (0.53-0.58) | 0.49*** | (0.45-0.53) | 0.48*** | (0.40-0.57) | 0.65*** | (0.64-0.67) | 0.67*** | (0.62-0.73) |

*p < 0.05 **p < 0.01 ***p < 0.001

**Appendix Table 3. Unadjusted Two-Part Model Results of Having Any Health Care Costs and Per Member Per Month Cost Models among New Medicaid Enrollees Pre-COVID and New Enrollees During COVID, North Carolina**

| *Covariate* | *Any Cost Accrued* | | *Exponentiated Beta (SE)* |
| --- | --- | --- | --- |
| Enrollment Month | *RR* | *(95% CI)* |  |
| January | Reference | | Reference |
| February | 1.02* | (1.00-1.04) | 1.02 (1.03) |
| March | 0.80*** | (0.78-0.82) | 1.09*** (1.03) |
| April | 0.92*** | (0.90-0.94) | 1.02 (1.03) |
| May | 0.97*** | (0.95-0.99) | 1.04 (1.03) |
| June | 0.95*** | (0.93-0.97) | 1.03 (1.03) |
| July | 0.94*** | (0.92-0.96) | 1.07** (1.03) |
| August | 0.90*** | (0.88-0.92) | 1.00 (1.03) |
| September | 0.97** | (0.95-0.99) | 1.10*** (1.03) |
| October | 0.93*** | (0.91-0.95) | 1.06* (1.03) |
| November | 0.81*** | (0.79-0.82) | 1.12*** (1.03) |
| December | 0.88*** | (0.86-0.90) | 1.18*** (1.03) |
|  |  |  |  |
| Enrolled During COVID | 0.69*** | (0.68-0.71) | 0.86*** (1.02) |

*p < 0.05 **p < 0.01 ***p < 0.001

**Appendix Table 4. Unadjusted Models of Health Care Use and of New North Carolina Medicaid Enrollees During COVID Compared to New Enrollees Pre-COVID, Stratified by Eligibility Pathway**

|  | *Any ED* | | *Any*  *Nonemergent ED* | | *Any Potentially-*  *avoidable Hospitalization* | | *Any Primary care* | | *Any Dental* | | *Any Cost Accrued* | |
| --- | --- | --- | --- | --- | --- | --- | --- | --- | --- | --- | --- | --- |
| *Eligibility Pathway* | *RR* | *(95% CI)* | *RR* | *(95% CI)* | *RR* | *(95% CI)* | *RR* | *(95% CI)* | *RR* | *(95% CI)* | *RR* | *(95% CI)* |
| ABD, MQBQ, MQBB, MQBE | 0.57*** | (0.53-0.62) | 0.55*** | (0.47-0.65) | 0.54*** | (0.41-0.72) | 0.56*** | (0.52-0.60) | 0.54*** | (0.43-0.68) | 0.75*** | (0.72-0.77) |
| Pregnant Women & BCC | 0.64*** | (0.59-0.70) | 0.53*** | (0.43-0.65) | 0.80 | (0.34-1.86) | 0.77*** | (0.74-0.79) | 0.59** | (0.42-0.82) | 0.87*** | (0.85-0.88) |
| Low-Income Adult | 0.55*** | (0.52-0.57) | 0.50*** | (0.45-0.55) | 0.45*** | (0.36-0.57) | 0.66*** | (0.63-0.69) | 0.66*** | (0.61-0.73) | 0.67*** | (0.65-0.68) |
| General Pediatrics | 0.61*** | (0.54-0.69) | 0.45*** | (0.34-0.61) | 0.74 | (0.41-1.34) | 0.70*** | (0.63-0.78) | 0.74* | (0.58-0.93) | 0.71*** | (0.66-0.75) |
| Other | 0.52*** | (0.46-0.58) | 0.45*** | (0.32-0.62) | 0.56* | (0.34-0.91) | 0.74*** | (0.69-0.80) | 0.48** | (0.31-0.76) | 0.81*** | (0.78-0.83) |

*p < 0.05 **p < 0.01 ***p < 0.001

**Appendix Table 5. Adjusted Models of Health Care Use and Costs of New North Carolina Medicaid Enrollees During COVID Compared to New Enrollees Pre-COVID, Stratified by Eligibility Pathway**

|  | *Any ED* | | *Any*  *Nonemergent ED* | | *Any Potentially-*  *avoidable Hospitalization* | | *Any Primary care* | | *Any Dental* | | *Any Cost Accrued* | |
| --- | --- | --- | --- | --- | --- | --- | --- | --- | --- | --- | --- | --- |
| *Eligibility Pathway* | *RR* | *(95% CI)* | *RR* | *(95% CI)* | *RR* | *(95% CI)* | *RR* | *(95% CI)* | *RR* | *(95% CI)* | *RR* | *(95% CI)* |
| ABD, MQBQ, MQBB, MQBE*^1^* | 0.61*** | (0.56-0.67) | 0.59*** | (0.49-0.70) | –^2^ |  | 0.59*** | (0.55-0.63) | 0.52*** | (0.41-0.67) | 0.74*** | (0.72-0.77) |
| Pregnant Women & BCC | Adjusted models had major convergence issues within this subgroup | | | | | | | | | | | |
| Low-Income Adult*^1^* | 0.57*** | (0.54-0.61) | 0.52*** | (0.46-0.58) | –^2^ |  | 0.69*** | (0.66-0.72) | 0.69*** | (0.62-0.76) | 0.70*** | (0.68-0.73) |
| General Pediatrics^3^ | 0.57*** | (0.50-0.66) | 0.44*** | (0.32-0.60) | –^2^ |  | 0.68*** | (0.61-0.77) | 0.76* | (0.58-0.99) | 0.68*** | (0.63-0.72) |
| Other^4^ | 0.62*** | (0.53-0.71) | 0.56** | (0.38-0.82) | –^2^ |  | 0.71*** | (0.66-0.77) | 0.46** | (0.29-0.73) | 0.81*** | (0.79-0.84) |

*p < 0.05 **p < 0.01 ***p < 0.001

^1^ Adjusted for month of enrollment, rural/urban, race, ethnicity, sex, and age

^2^ Did not converge due to too few cases of the specified outcome among these new enrollees

^3^Adjusted for month of enrollment, rural/urban, race, ethnicity, and sex
^4^Adjusted for month of enrollment, rural/urban, race, ethnicity, sex, and age; except race and ethnicity were not included in the dental model due to convergence issues.

**Appendix Table 6. Unadjusted and Adjusted Two-Part Model Results of Per Member Per Month Cost Models of New North Carolina Medicaid Enrollees During COVID Compared to New Enrollees Pre-COVID, Stratified by Eligibility Pathway**

|  | *Unadjusted* | *Adjusted* |
| --- | --- | --- |
| *Eligibility Pathway* | *Exponentiated Beta (SE)* | *Exponentiated Beta (SE)* |
| ABD, MQBQ, MQBB, MQBE^1^ | 1.16** (1.05) | 1.15** (1.05) |
| Pregnant Women & BCC^1^ | 0.87*** (1.02) | 0.89*** (1.03) |
| Low-Income Adults^1^ | 0.85*** (1.02) | 0.86*** (1.03) |
| General Pediatrics^2^ | 0.89* (1.06) | 0.88* (1.07) |
| Other^1^ | 0.91 (1.07) | 0.90 (1.07) |

*p < 0.05 **p < 0.01 ***p < 0.001

^1^ Adjusted for month of enrollment, rural/urban, race, ethnicity, sex, and age

^2^ Adjusted for month of enrollment, rural/urban, race, ethnicity, and sex

**Figures 1 – 12. Monthly Trends in Health Care Use and Costs among New North Carolina Medicaid Enrollees Before and During COVID**
